# Triple-negative breast cancer: understanding Wnt signaling in drug resistance

**DOI:** 10.1186/s12935-021-02107-3

**Published:** 2021-08-10

**Authors:** Parnaz Merikhian, Mohammad Reza Eisavand, Leila Farahmand

**Affiliations:** Recombinant protein department, Breast Cancer Research Center, Motamed Cancer Institute, ACECR, 146, South Gandhi Ave., Vanak Circus, Tehran, Iran

**Keywords:** Triple negative breast cancers (TNBCs), Wnt/β-catenin, Drug resistance, Tumorigenesis, Combination therapy

## Abstract

Triple-negative breast cancer (TNBC) is not as prevalent as hormone receptor or HER2-positive breast cancers and all receptor tests come back negative. More importantly, the heterogeneity and complexity of the TNBC on the molecular and clinical levels have limited the successful development of novel therapeutic strategies and led to intrinsic or developed resistance to chemotherapies and new therapeutic agents. Studies have demonstrated deregulation of Wnt/β-catenin signaling in tumorigenesis which plays decisive roles at the low survival rate of patients and facilitates resistance to currently existing therapies. This review summarizes mechanisms of Wnt/β-catenin signaling for resistance development in TNBC, the complex interaction between Wnt/β-catenin signaling, and the transactivated receptor tyrosine kinase (RTK) signaling pathways, lymphocytic infiltration, epithelial-mesenchymal transition (EMT), and induction of metastasis. Such associations and how these pathways interact in the development and progression of cancer have led to the careful analysis and development of new and effective combination therapies without generating significant toxicity and resistance.

## Background

Triple-negative breast cancer (TNBC) cells are identified by the lack of receptor targeted therapies including human epidermal receptor 2 (HER2), estrogen receptor (ER) and progesterone receptor (PR), and are major cause of lack of better therapies, consisting about 15–20% of recently diagnosed breast cancer [1]. TNBC tumors are mostly difficult to currently existing therapies due to distinct molecular profile. High mitotic rate and enhanced lymphocytic infiltration. Based on biomarker-driven therapeutic approaches, TNBCs are categorized into luminal androgen receptor, immune-enriched, PI3K/Akt/mTOR activated and DNA repair deficiency [2].

Based on tumors’ characteristics including size, morbidity, and the number of involved lymph nodes, chemotherapeutic agents have been the primary systemic options for TNBC patients [3]. However, resistance to chemotherapeutic agents, such as anthracyclines, taxanes, capecitabine, gemcitabine, eribulin as well as biomarker-based treatments and immune checkpoint inhibitor-based immunotherapy mostly occurs and causes limited results or no response to the treatments. Recent studies have revealed various molecular mechanisms and signaling pathways concerning proliferation, metabolism, survival, and movement in TNBC, which consequently lead to the resistance to novel targeted therapies [4, 5]. Therefore, it is of importance to elucidate the molecular factors behind intrinsic and acquired resistance to restore chemo- and targeted therapies.

**Fig. 1:**
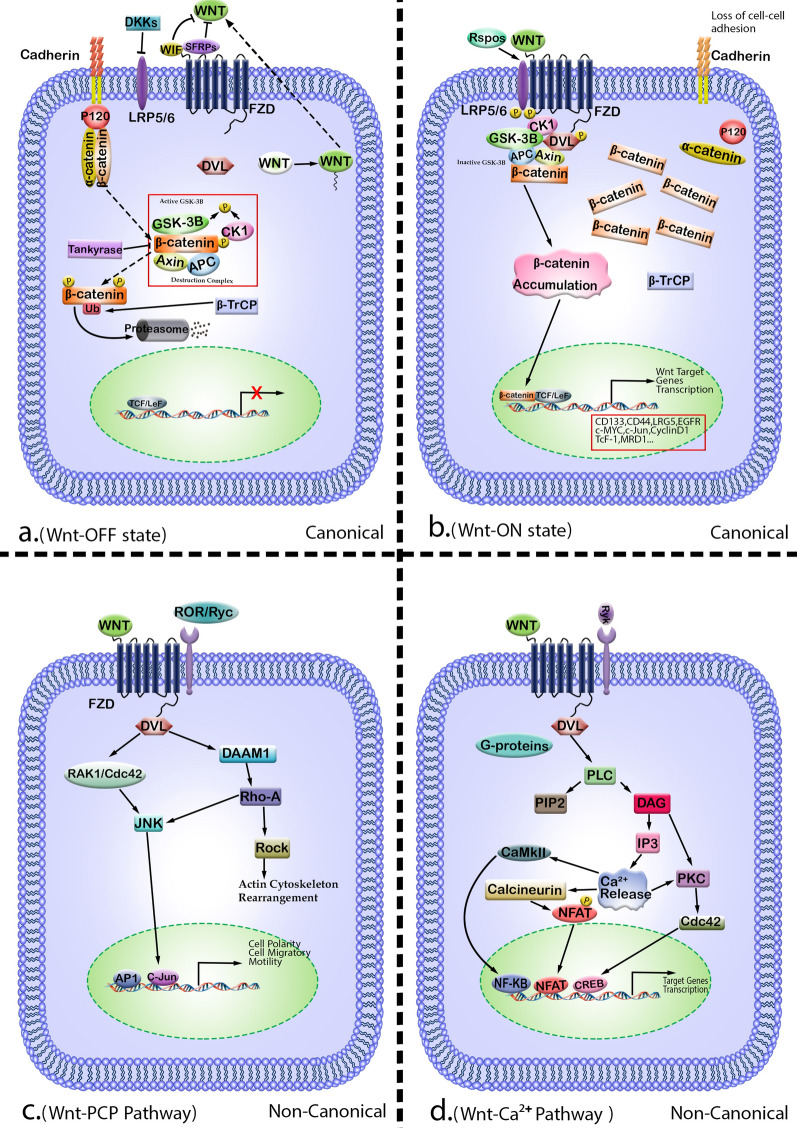
An overview of the Wnt signaling pathway. **a** In the absence of Wnt ligands (Wnt-Off state), β-catenin is released from the cytomembrane, sequestered in a destructive protein complex, that is composed of adenomatous polyposis coli (APC), the scaffolding protein axin, casein kinase 1 (CK1), and glycogen synthase kinase 3β (GSK-3β). The Dkks, WIF, and SFRPs act as antagonists. The phosphorylations by CK1 and GSK-3β recruit β-propeller domain of the E3 ubiquitin ligase (β TrCP) and subsequently cause the β catenin proteasomal degradation and transcriptional repression of Wnt target genes. **b** Canonical Wnt/β-catenin signaling is activated by binding of Wnt ligands (Wnt-On state) to a receptor complex composed of FZD and LRP 5/6. The recruitment of phosphorylated DVL to FZD inhibits the APC/CK1/GSK-3β destruction complex and blockade of β-catenin by GSK-3β. Accumulated β-catenin in the cytoplasm translocate into the nucleus, where it regulates target gene expression with the Tcf/Lef family of transcription factors. **c** In Wnt planar cell polarity (Wnt-PCP) signaling, Wnt binds multiple receptors including FZD and co-receptors ROR and Ryk. This activates Rho-A and RAK1/Cdc42, which activate ROCK and JNK (c-Jun N-terminal kinase), respectively, leading to actin cytoskeleton rearrangement and cell polarity through AP-1. **d** In ON-state non-canonical Wnt/Ca2+ signaling pathway, the binding of Wnt promotes FZD-mediated activation of G proteins and Ryk and initiates the release of Ca2+ from intracellular stores and activation of Ca2+-dependent effector molecules. Several Ca2+-sensitive targets, i.e., PKC, CamKII, and calcineurin, have been identified as downstream of the Wnt/Ca2+ pathway

Wnt ligands activate distinct intracellular signaling, which can be categorized into β-catenin-dependent and β-catenin-independent pathways (Fig. 1). According to current researches, deregulation of the Wnt signaling pathway is related to tumor propagation, invasion, and development of resistance to anticancer agents which steer tumors toward a malignant form of progression, and ultimately leading to poor prognosis of overall survival [6, 7]. Interestingly, recent findings in TNBC tumors have elucidated the crosstalk between the Wnt pathway and multiple growth factors and developmental signaling pathways and β-catenin as a main downstream effector of the pathway at multiple levels [8]. Epidermal growth factor receptor (EGFR), transforming growth factor β receptor (TGFβ-R), insulin/IGF receptor (IGF-R), and vascular endothelial growth factor receptor (VEGFR) are among the most important RTKs presented in TNBC, and since they are the converging point of many intracellular signaling pathways, one can suggest that deregulation of Wnt/β-catenin can be considered as one of the main reasons for RTK inhibitors not inducing desired responses in clinical trials [9, 10]. Our previous data illustrated that the major effector of the canonical Wnt pathway, β-catenin, is stabilized in tumors primarily via Wnt ligand overexpression, down-regulation of Wnt ligand antagonists, or loss of the APC tumor suppressor. As a consequence of its stabilization, β-catenin translocates to the nucleus, where it controls gene expression through its association with members of the T cell factor (TCF) family of transcription factors. Some of the β-catenin/TCF transcriptional targets implicated in tumor initiation and progression include cell cycle regulators cyclin D1 and c-Myc [11]. Indeed, identifying the correlation between Wnt signaling and different carcinogenic pathways and their interaction with the heterogeneity of TNBC may represent a better comprehension in the development of novel therapeutic approaches for patients. Based on this brief preface, here, we review the role of Wnt signaling in mechanisms of resistant development in TNBC cells and then investigate the crosstalks between multiple developmental signaling pathways and the Wnt pathway and finally suggest how multi-targeting of these tumor-associated pathways are highly synergistic with currently existing therapeutic agents.

### Wnt signaling associated with the development of resistance to anti-cancer therapies

Although many reports have suggested that TNBCs tend to respond well to chemotherapeutic drugs, patients may frequently develop a chemorefractory and poor prognosis. Interestingly, recent findings lend support to the possibility that targeting Wnt/β-catenin signaling in combination with chemo- and targeted- therapies in patients with TNBC exhibits highly synergistic results and prevent Wnt signaling function in chemoresistant cancer cells. According to the in vitro studies of Xu et al. [12], β-catenin expression correlates with chemoresistance of TNBC as β-catenin knockdown resensitized TNBC cells to doxorubicin or cisplatin-mediated cell death. However, it still needs further investigations to consider promising therapeutic targets of the Wnt pathway and its components in TNBC.

Several mechanisms can lead to the development of chemoresistance, among which ATP-binding cassette (ABC) transporters are the most thoroughly validated. Three transporters have been extensively identified in TNBC comprising (i) multidrug-resistant protein-1 and -8 (MRP1 and 8), (ii) breast cancer resistance protein (ABCG2), and (iii) the P-glycoprotein (MDR1) pump which pumps a wide array of chemotherapeutics out of the cancer cells. Previous studies have demonstrated increased nuclear and cytoplasmic β-catenin accumulation induced ABCB1 (MDR1 or P-gp) in TNBC involving *BRCA1/2* mutation to excrete Poly (ADP-ribose) polymerase (PARP) inhibitors (PARPi) actively out of the cells [13]. Therefore, combination therapies targeting the Wnt/β-catenin pathway to overcome PARP inhibitor resistance can bring novel opportunities to improve clinical responses. As reported by Zhang et al. [14], frizzled receptor 1 (FZD1) and P-gp overexpression increased the doxorubicin-resistant in breast cancer cells. Their results indicated that silencing the *FZD1* gene expression, by siRNA, reduced cytoplasmic/nuclear β-catenin level and P-gp overexpression.

Many studies have shown that cancer stem cells (CSCs) are more resistant to conventional therapies in TNBC, which cause survival and relapse of cancer. Moreover, it has been shown that hypoxia inducing factor 1-α (HIF-1α) enhances Wnt/β-catenin signaling in the hypoxic microenvironment and promotes β-catenin stabilization and upregulation of CSCs transcription genes [15]. However, there has been no direct evidence to establish such a relation in TNBC. Notably, Wnt signaling causes tumor therapeutic resistance via the synergistic interaction between Wnt target gene c-MYC and HIF-1α which can subsequently attenuate the responsiveness of cancer cells to the drugs combating them.

These observations were supported by the elevated activation of the Wnt signaling pathway following stimulation of drug inactivation/detoxification, deregulation of DNA damage repair pathways, cell cycling, and apoptosis. Consequently, as Wnt signaling becomes aberrantly activated in cancer cells, these signaling mechanisms become perturbed resulting in the development of resistance to selected TNBC’s therapeutic agents. Therefore, there is an urgent clinical need to better understand diverse mechanisms of resistance to multiple conventional and targeted cancer therapies through elevated Wnt signaling and to introduce novel combinatorial therapies extending TNBC disease-free intervals.

### Wnt pathway association with Tumor propagation and signaling transduction pathways

So far, multiple crosstalks between Wnt signaling and other intracellular signaling pathways have been identified in TNBC, which can cause resistance to therapies. The gene-expression profiles of TNBC cells have shown an association between EGFR and Wnt signaling in TNBC patients treated in the adjuvant setting. Recent findings on the mechanisms of resistance in TNBC cells, which constitutively overexpress EGFR, demonstrate that silencing Wnt/β-catenin signaling contributes to the reduction of the levels of β-catenin, RAS, and EGFR and put forward the possibility of combinatorial treatment for TNBC patients [16]. Given the fact that β-catenin localizes in adherens junctions and these structures are normally maintained by kinases, reduction of membrane β-catenin in EGFR-overexpressing tumors might be a confirmation of EGFR-induced destabilization of cell-to-cell adhesion [17]. In addition, adequate evidence has shown that EGFR can directly phosphorylate β-catenin and stimulate EMT by disturbing the interaction between β-catenin and E-cadherin in tumor cells, leading to enhanced tumor spread [18].

**Fig. 2:**
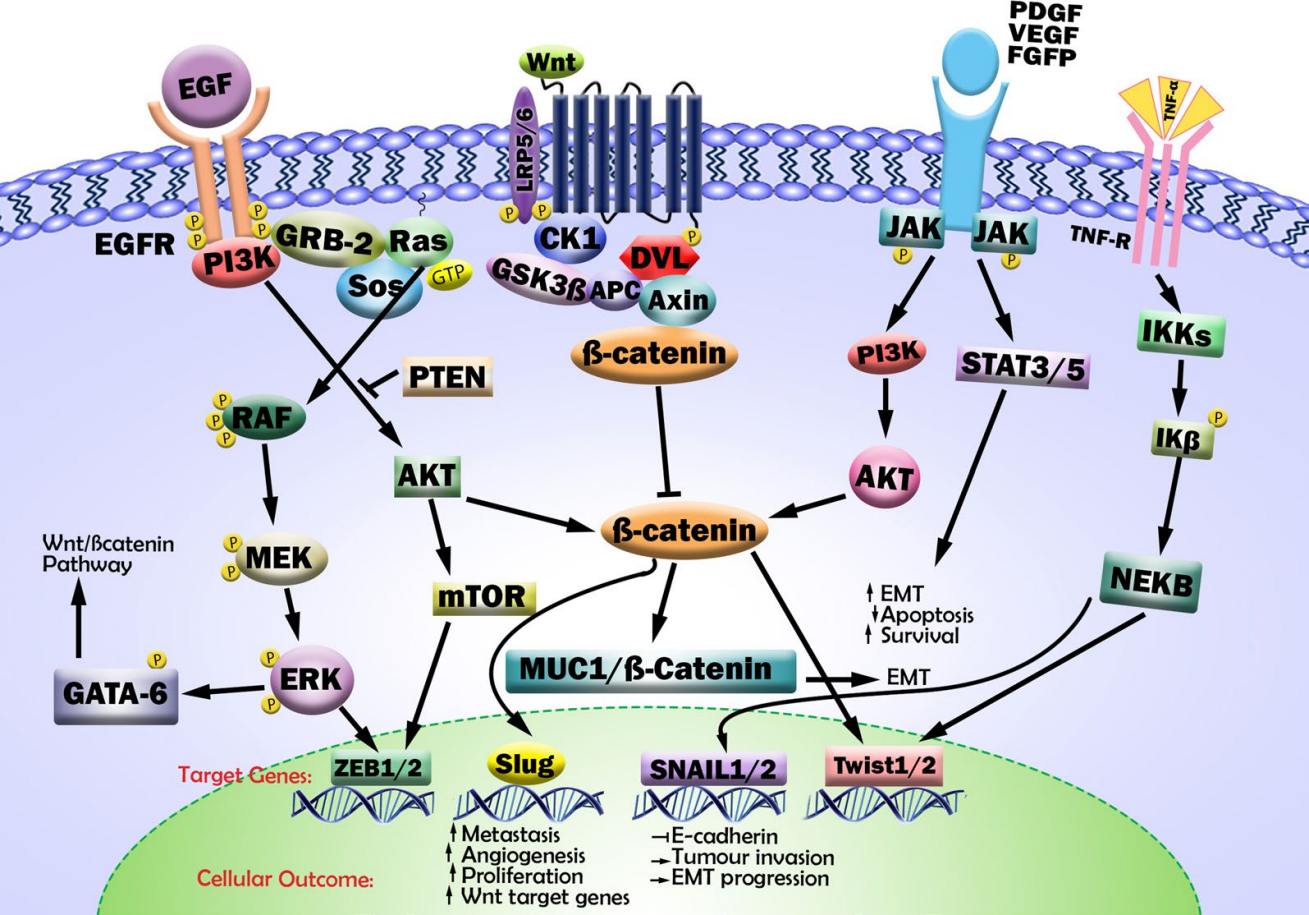
Cross-talk of the Wnt/β-catenin pathway with the RTK family receptors and its role in the induction of EMT. The Wnt/β-catenin and EGFR signaling pathways, on the binding of specific ligands, can activate each other. The binding of Wnt ligands with FZD receptors transactivates EGFR signaling by MMP-mediated release of EGF ligands. In turn, EGFR signaling transactivates the Wnt/β-catenin pathway through the PI3K/Akt and Ras/Raf/MEK/Erk signaling cascades. Akt can induce β-catenin by triggering its nuclear translocation or blocking GSK-3β activities. PTEN, which acts as a tumor suppressor and inhibits the activation of Akt, also negatively regulates β-catenin nuclear translocation. In addition, the aberrant activation of the EGFR pathway leads to an increase in free β-catenin accumulation in the cytoplasm through inducing dissociation from α-catenin. Several cell signaling pathways induce the expression of EMT-inducing transcription factors such as ZEB, SNAIL, and TWIST to push the tumor cells toward proliferation and metastasis. Moreover, the binding of several growth factors and cytokines to their receptors acts to induce the phosphorylation and activation of JAKs and activator of transcription proteins (STATs); STAT3/5 dimers stimulate the transcription of genes encoding EMT transcription factors, anti-apoptotic and survival proteins. In addition, in response to stimuli, such as TNF-α, the IKK complex is activated, resulting in phosphorylation of IKB and its degradation by the proteasome, and allowing the translocation of NF-κB into the nucleus

The interaction between EGFR signaling activation and the Wnt/β-catenin pathway involves series of kinase signaling cascades for the interruption of adherent junctions. Most important examples include crosstalk between Wnt/β-catenin and EGF/RAS/ERK signaling pathways which involve accelerating β-catenin nuclear translocation through GSKGSA-3β inhibition [19, 20]. Conversely, Wnt/β-catenin signaling can regulate RAS stability by reducing proteasomal ubiquitination and degradation. RAS stabilization at plasma membrane activates the RAF/MEK/ERK (MAPK) signaling cascade by series of phosphorylations and triggers transcription factors activations leading to hyperproliferation, high cancer stem cells (CSCs) activation, and metastasis [21] (Fig. 2).

Wnt signaling confers EGFR-dependent activation of the PI3K/Akt/mTOR pathway and nucleus localization of β-catenin and EGFR target genes. Akt can activate β-catenin nuclear translocation and reduce GSK-3β activities, which results in increased transcription of proliferative genes, and tumor cell invasion [22]. In addition, It has been indicated that reduced expression of PTEN, a tumor suppressor by negatively regulating the Akt signaling, mediates translocation of β-catenin to the nucleus and defines a subclass of highly lethal TNBCs with PTEN-low that should be prioritized for aggressive therapy [23].

Recent evidence published by Solzak et al. has indicated that mono-agent therapies (using PI3K inhibitors) are commonly subject to resistant development in the majority of TNBCs. As such, combination therapy against both PI3K and Wnt pathways using buparlisib (panPI3K) and Wnt974 (Wnt-pathway), demonstrates significant in vitro and in vivo synergy against TNBC cell lines and xenografts [24]. Mentioning this point also seems critical that the Wnt/β-catenin pathway has been implicated in the maintenance of resistance to PI3K inhibitors, and it seems the use of β-catenin inhibitors may resensitize PIK3CA (Phosphatidylinositol-4,5-Bisphosphate 3-Kinase Catalytic Subunit Alpha) to PI3K inhibitors in patients with PIK3CA-mutated TNBC [25, 26]. This indicates the regulation of cancer cell growth in relation to increasing tumor cell sensitivity to combination therapeutic agents. In certain settings, the association between Wnt pathway activation, tumor-related signaling pathways, and even epigenetic effects could contribute to a multidrug-resistance phenotype in advanced TNBC. Thus, identifying the Wnt signaling network, which so far does not have any approved targeted therapy, is emphasized to implement optimal efficacy in our current treatments.

In addition, the vast majority of studies have focused on the Wnt/ROR signaling and its influence on tumor cell function starting with two receptor tyrosine kinase-like orphan receptors (RORs), ROR1 and ROR2 with the activation of non-canonical Wnt signaling responses triggered by binding of Wnt5A ligand [25, 27]. Recent findings have suggested the role of Wnt5A/ROR1 signaling in the progression of TNBC tumor cells through the phosphorylation of ROR1 by several kinases which results in the inhibition of anti-apoptotic pathways and activation of pro-survival signalings, such as Wnt/PCP, MAPK/ERK, PI3K/Akt/mTOR, TGF-β/SMAD and NF-κB. As a result of these mechanisms, the Wnt5A/ROR1 signaling axis plays a negative role associated with enhanced tumor cell migration or induces a transcriptional response leading to the expression of genes that contribute to cell proliferation, survival, EMT, or therapy resistance [28, 29]. Undoubtedly, the ongoing development of various strategies targeting either Wnt5A or the Wnt5A/ROR1 is surely helpful for TNBC therapy.

### Wnt signaling and the tumor microenvironment

Performed studies on TNBC cells have shown the biological importance of tumor-infiltrating lymphocytes (TILs) and the balance between cytotoxic and regulatory pathways in the tumor microenvironment. It has previously shown that the presence of TILs in the tumor site, in early-stage TNBC correlated with significantly improved clinical outcomes and disease-free and overall survival rates in patients [30]. Aberrant intratumoral Wnt/β-catenin signaling activation correlates with the hypoxic microenvironment of TNBC tumors, immune suppression, and excluding T cell infiltration, which results in immune escape by tumors and limits treatment responsiveness [31]. Recent evidence has indicated that the distinct infiltrating cell types have prognostic significance in breast cancer. A high level of CD8+ (cytotoxic T) lymphocyte infiltration, indicates better patient survival and responses to therapies in basal-like breast cancer, while high density of forkhead box P3 (FoxP3+) regulatory T cells (T-Regs) are frequently linked to poor survival outcomes [32]. These data have also proposed that activation of TCF-1/β-catenin signaling can result in stem cell-like phenotypes resulting in the formation of memory CD8 + T cells and then the differentiation of naïve CD8+ T cells into CD8+ T effector cells is inhibited. By preventing the infiltration of effector CD8 + T cells during tumor progression, tumor cells evade immune elimination [33]. Furthermore, Dai et al. [34] discovered that Wnt/β-catenin signaling limits the antitumor immune response resulting in the infiltration of T-regs into the tumor microenvironment (TME). Consistent with this finding they demonstrated that the production of negative immunomodulators FoxP3 can be reduced by blocking the Wnt/β-catenin signaling pathway in T-Regs. These pieces of evidence significantly suggest that further investigations concerning the anti-tumor effects of TILs and its connection with oncogenic Wnt/β-catenin activities are required to consider Wnt modulator for use in combination immunotherapy regimens for TNBC.

The interplay between positive and negative immunoregulatory signals is carried on to ensure that the adaptive immune system is capable of defending the host while maintaining self-tolerance and preventing autoimmunity and one important aspect is the PD-1/PD-L1 axis. PD-1 and PD-L1 are immune check-point ligand and receptor which restrict T cell effector functions within tissues [35]. TNBC cells with overexpressed PD-1 show blockade of the antitumor immune effects which lead to cancer metastasis by induced EMT [36]. Therapeutic agents, with inhibitory effects on PD-1 and PD-L1, seem to hold great promise as a novel approach in cancer treatment, particularly in combination with other drugs. Although the mechanisms underlying resistance to checkpoint inhibition in TNBC immunotherapy remain obscure, delineating Wnt signaling in relation to drug resistance is of crucial importance, especially for the development of a new therapeutic strategy for patients who remain at high risk for recurrence.

In addition, increasing studies indicated up-regulation of CSC-related Wnt signaling pathway and tumor-intrinsic activation of β-catenin signaling in PD-L1High TNBCs [37, 38]. These findings suggested treatment of TNBC patients with selective Wnt/β-catenin inhibitors which can downregulate drug resistance and resensitize TNBC to anti-PD-L1/anti-CTLA-4 monoclonal antibody immunotherapy. In succession, several trials are also exploring Wnt/β-catenin inhibitors administered in combination with various immunochemotherapy drugs.

### Wnt signaling association with tumor cell migration and metastasis

**Fig. 3:**
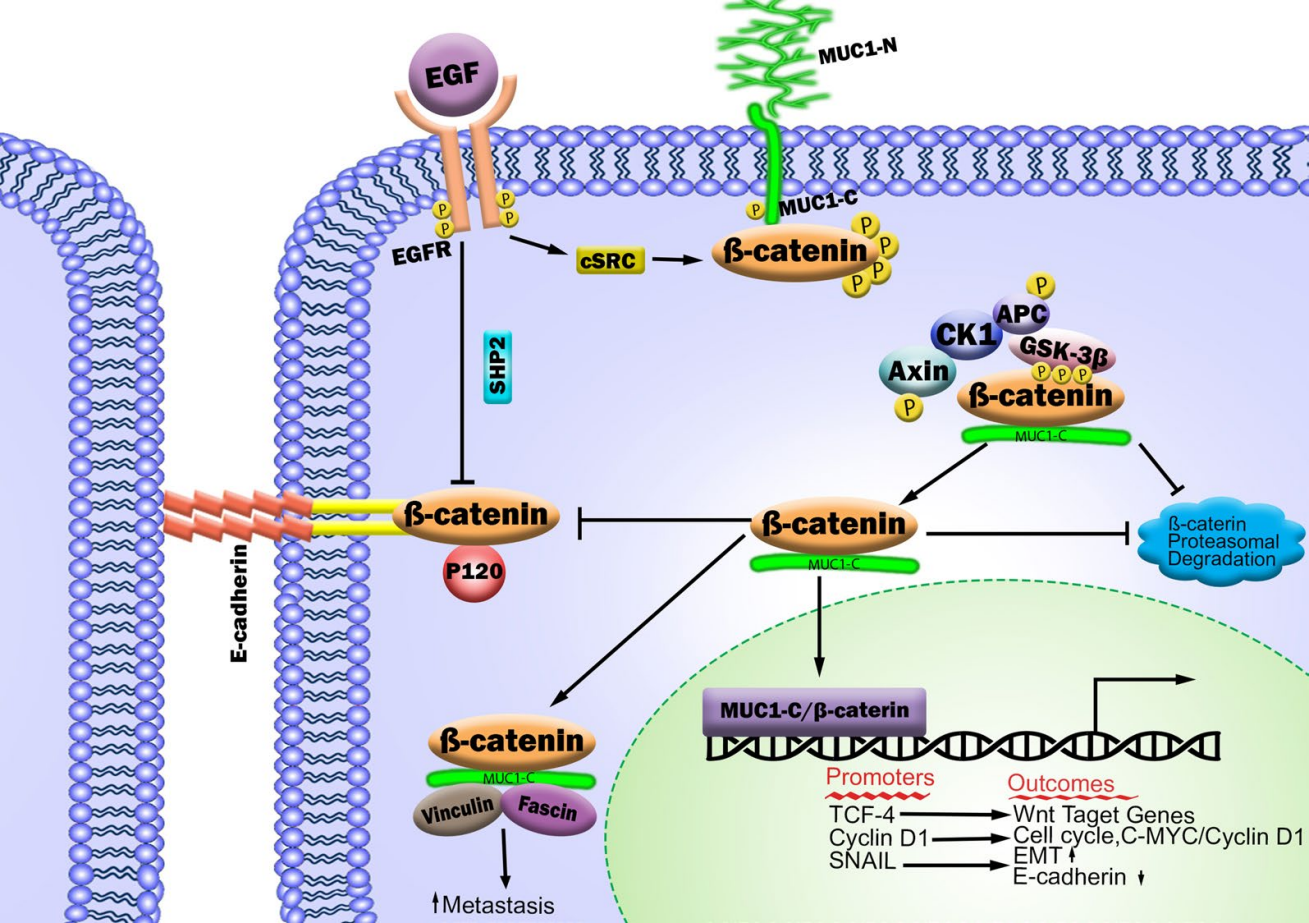
Schematic overview of β-catenin–MUC1 dynamics. MUC1 interacts with various receptor tyrosine kinases, such as EGFR, FGFR, PDGFR, and HER2. When the serine-rich domain of MUC1-C is phosphorylated by EGFR or cSRC, the affinity for β-catenin binding is increased. Following cleavage of the cytoplasmic domain, the MUC1/β- catenin complex localizes adjacent to the membrane and binds cytoskeleton members (fascin and vinculin), or competitively binds with E-cadherin to prevent E-cadherin/β-catenin complex formation. The formation of the MUC1/β-catenin complex stabilizes β-catenin in the cytoplasm by preventing its phosphorylation-mediated proteasomal degradation. In addition, MUC1-CD/β-catenin is translocated into the nucleus and interacts with (TCF7L2/TCF4) transcription factors to activate Wnt-triggered genes transcription

Cancer cell dissemination and metastasis are well-established as the worst trait of cancer progression and are correlated with therapeutic resistance. Loss of E-cadherin and accumulation of nuclear β-catenin induce CD44^high^/CD24^low^ stem-like cells in a set of mammary cancer cell lines [39]. Considering the importance of the Wnt pathway in stem cell biology, it is not surprising that aberrant Wnt/β-catenin signaling has been implicated in the tumorigenic potential of CSCs and metastasis. In addition, aberrant Wnt signaling through up-regulated frizzled-7 (FZD7) receptor is associated with tumorigenesis and poor prognosis [40, 41]. As such, Guanman et al. [42] revealed that the FZD7-induced canonical Wnt/β-catenin pathway may contribute to EMT progression and metastasis. They indicated that blocking endogenous FZD7 caused an increase of E-cadherin expression level and decrease of mesenchymal cells markers expression, such as vimentin, fibronectin, N-cadherin, snail, and MMP7 compared with their respective control cells. Furthermore, mucin 1 (MUC1) overexpression in tumor cells can suppress normal E-cadherin function and cell adhesion [43]. In addition, the MUC1-CD/β-catenin complex can either (i) localize adjacent to the cell membrane and bind to cytoskeleton members Fascin and Vinculin to stabilize β-catenin and prevent interaction between β-catenin and E-cadherin, or (ii) translocate into the nucleus, where it aids nuclear co-factor TCF4 for Wnt/β-catenin target genes transcription (Fig. 3). Furthermore, it has been proved that that GSK-3β has interaction with MUC1 in a manner that blocks MUC1/β-catenin complex formation [44]. As mentioned, GSK3β phosphorylates β-catenin and targets it for proteasomal degradation. MUC1 overexpression blocks GSK3β-mediated phosphorylation and degradation of β-catenin, thereby leading to the up-regulation of β-catenin level [45, 46]. Taken together, these findings have provided compelling support for MUC1-CD-induced Wnt signaling through activation of β-catenin/TCF/LEF pathway and transcription of Wnt target genes.

## Conclusion and future direction

TNBC is extensively studied over the last few decades on account of its unique clinical pathologies, such as larger tumor size, nodal involvement, high distant recurrence rates, and molecular features. Currently, targeted therapy in combination with chemotherapy is approved to treat locally advanced or metastatic TNBCs. Therefore, screening more reliable biomarkers is imperative, and understanding the signaling pathways that regulate biological behaviors may facilitate the establishment of effective therapeutic strategies. Wnt/β-catenin signaling’s crosstalk with TNBC-associated tumorigenic mechanisms results in amplification of their underlying signaling cascades and development of resistance to different therapeutic agents. Consequently, establishing new promising therapeutic regimens with specific targeting abilities of its upstream and downstream signaling seems to be an attractive challenge for overcoming resistance and prevention of cancer progression. Studies in preclinical and clinical trials have shown that the concurrent inhibition of Wnt signaling together with administration of cancer chemo- and targeted- therapies develop synergistic effects in the treatment of TNBCs. Despite several signs of progress in understanding the contributing role of Wnt signaling in the development of TNBC and possible mechanisms underlying the development of resistance to anti-cancer agents, still, many questions remain unsolved which must be answered in near future to precisely design safe combinational therapeutic strategies with high potency and low toxicity.

## Data Availability

Not applicable.

## Abbreviations

ABC: ATP-binding cassette
Akt: RAC-alpha serine/threonine-protein kinase
CSC: Cancer stem cell
EGFR: Epidermal growth factor receptor
ER: Estrogen receptor
FZD: Frizzled
HIF-1a: Hypoxia inducing factor 1a
IGF-R: Insulin growth factor receptor
MRP: Multidrug-resistant protein
MUC1: Mucin 1
PARP: Poly ADP-ribose polymerase
PI3K: Phosphatidylinositol 3-kinase
PIK3CA: Phosphatidylinositol-4,5-bisphosphate 3-kinase catalytic subunit alpha
PR: Progesterone receptor
P-gp: P-glycoprotein
siRNA: Small interfering RNA
TCF: T cell factor
TGFBR: Transforming growth factor receptor
TIL: Tumor-infiltrating lymphocyte
TNBC: Triple-negative breast cancer
T-regs: Regulatory T cells
VEGFR: Vascular endothelial growth factor receptor

## References

[1] Yao H, He G, Yan S, Chen C, Song L, Rosol TJ (2017). Triple-negative breast cancer: is there a treatment on the horizon?. Oncotarget.

[2] Marra A, Trapani D, Viale G, Criscitiello C, Curigliano G (2020). Practical classification of triple-negative breast cancer: intratumoral heterogeneity, mechanisms of drug resistance, and novel therapies. NPJ Breast Cancer.

[3] Marra A, Viale G, Curigliano G (2019). Recent advances in triple negative breast cancer: the immunotherapy era. BMC Med.

[4] Montor WR, Salas AROSE, de Melo FHM (2018). Receptor tyrosine kinases and downstream pathways as druggable targets for cancer treatment: the current arsenal of inhibitors. Mol Cancer.

[5] Qi F, Qin WX, Zang YS (2019). Molecular mechanism of triple–negative breast cancer–associated BRCA1 and the identification of signaling pathways. Oncology letters.

[6] Martin-Orozco E, Sanchez-Fernandez A, Ortiz-Parra I, Nicolas A-S (2019). WNT signaling in tumors: the way to evade drugs and immunity. Frontiers in immunology.

[7] Farahmand L, Darvishi B, Majidzadeh AK, Madjid Ansari A (2017). Naturally occurring compounds acting as potent anti‐metastatic agents and their suppressing effects on Hedgehog and WNT/β‐catenin signalling pathways. Cell Prolifer.

[8] Xu X, Zhang M, Xu F, Jiang S (2020). Wnt signaling in breast cancer: biological mechanisms, challenges and opportunities. Molecular Cancer.

[9] Butti R, Das S, Gunasekaran VP, Yadav AS, Kumar D, Kundu GC (2018). Receptor tyrosine kinases (RTKs) in breast cancer: signaling, therapeutic implications and challenges. Mol Cancer.

[10] Khodabakhsh F, Merikhian P, Eisavand MR, Farahmand L (2021). Crosstalk between MUC1 and VEGF in angiogenesis and metastasis: a review highlighting roles of the MUC1 with an emphasis on metastatic and angiogenic signaling. Cancer Cell Int.

[11] Lecarpentier Y, Schussler O, Hébert J-L, Vallée A (2019). Multiple targets of the canonical WNT/β-catenin signaling in cancers. Frontiers in oncology.

[12] Xu J, Prosperi JR, Choudhury N, Olopade OI, Goss KH (2015). β-Catenin is required for the tumorigenic behavior of triple-negative breast cancer cells. PloS one.

[13] Gangrade A, Pathak V, Augelli-Szafran CE, Wei H-X, Oliver P, Suto M (2018). Preferential inhibition of Wnt/β-catenin signaling by novel benzimidazole compounds in triple-negative breast cancer. Int J Mol Sci.

[14] Zhang H, Zhang X, Wu X, Li W, Su P, Cheng H (2012). Interference of Frizzled 1 (FZD1) reverses multidrug resistance in breast cancer cells through the Wnt/β-catenin pathway. Cancer Lett.

[15] Xu W, Zhou W, Cheng M, Wang J, Liu Z, He S (2017). Hypoxia activates Wnt/β-catenin signaling by regulating the expression of BCL9 in human hepatocellular carcinoma. Sci Rep.

[16] Ryu W-J, Lee JD, Park J-C, Cha P-H, Cho Y-H, Kim JY (2020). Destabilization of β-catenin and RAS by targeting the Wnt/β-catenin pathway as a potential treatment for triple-negative breast cancer. Exp Mol Med.

[17] Lakis S, Dimoudis S, Kotoula V, Alexopoulou Z, Kostopoulos I, Koletsa T (2016). Interaction between beta-catenin and EGFR expression by immunohistochemistry identifies prognostic subgroups in early high-risk triple-negative breast cancer. Anticancer Res.

[18] Wang W, Pan Q, Fuhler GM, Smits R, Peppelenbosch MP (2017). Action and function of Wnt/β-catenin signaling in the progression from chronic hepatitis C to hepatocellular carcinoma. J Gastroenterol.

[19] Caspi M, Zilberberg A, Eldar-Finkelman H, Rosin-Arbesfeld R (2008). Nuclear GSK-3β inhibits the canonical Wnt signalling pathway in a β-catenin phosphorylation-independent manner. Oncogene.

[20] Lemieux E, Cagnol S, Beaudry K, Carrier J, Rivard N (2015). Oncogenic KRAS signalling promotes the Wnt/β-catenin pathway through LRP6 in colorectal cancer. Oncogene.

[21] Robertson H, Hayes JD, Sutherland C (2018). A partnership with the proteasome; the destructive nature of GSK3. Biochem Pharmacol.

[22] Moradi-Kalbolandi S, Hosseinzade A, Salehi M, Merikhian P, Farahmand L (2018). Monoclonal antibody-based therapeutics, targeting the epidermal growth factor receptor family: from herceptin to Pan HER. J Pharm Pharmacol.

[23] McCubrey JA, Steelman LS, Bertrand FE, Davis NM, Sokolosky M, Abrams SL (2014). GSK-3 as potential target for therapeutic intervention in cancer. Oncotarget.

[24] Wang D-Y, Gendoo DM, Ben-David Y, Woodgett JR, Zacksenhaus E (2019). A subgroup of microRNAs defines PTEN-deficient, triple-negative breast cancer patients with poorest prognosis and alterations in RB1, MYC, and Wnt signaling. Breast Cancer Res.

[25] Solzak JP, Atale RV, Hancock BA, Sinn AL, Pollok KE, Jones DR (2017). Dual PI3K and Wnt pathway inhibition is a synergistic combination against triple negative breast cancer. NPJ breast cancer.

[26] Lehmann BD, Bauer JA, Schafer JM, Pendleton CS, Tang L, Johnson KC (2014). PIK3CA mutations in androgen receptor-positive triple negative breast cancer confer sensitivity to the combination of PI3K and androgen receptor inhibitors. Breast Cancer Res.

[27] Menck K, Heinrichs S, Baden C, Bleckmann A (2021). The WNT/ROR pathway in cancer: from signaling to therapeutic intervention. Cells.

[28] Medina MA, Oza G, Sharma A, Arriaga L, Hernández Hernández JM, Rotello VM (2020). Triple-negative breast cancer: a review of conventional and advanced therapeutic strategies. Int J Environ Res Public Health.

[29] Lopez-Bergami P, Barbero G (2020). The emerging role of Wnt5a in the promotion of a pro-inflammatory and immunosuppressive tumor microenvironment. Cancer Metastasis Rev.

[30] Chien H-P, Ueng S-H, Chen S-C, Chang Y-S, Lin Y-C, Lo Y-F (2016). Expression of ROR1 has prognostic significance in triple negative breast cancer. Virchows Arch.

[31] Loi S, Drubay D, Adams S, Pruneri G, Francis PA, Lacroix-Triki M (2019). Tumor-infiltrating lymphocytes and prognosis: a pooled individual patient analysis of early-stage triple-negative breast cancers. J Clin Oncol.

[32] Li X, Xiang Y, Li F, Yin C, Li B, Ke X (2019). WNT/β-catenin signaling pathway regulating T cell-inflammation in the tumor microenvironment. Front Immunol.

[33] Pai SG, Carneiro BA, Mota JM, Costa R, Leite CA, Barroso-Sousa R (2017). Wnt/beta-catenin pathway: modulating anticancer immune response. J Hematol Oncol.

[34] Gattinoni L, Zhong X-S, Palmer DC, Ji Y, Hinrichs CS, Yu Z (2009). Wnt signaling arrests effector T cell differentiation and generates CD8+ memory stem cells. Nat Med.

[35] Dai W, Liu F, Li C, Lu Y, Lu X, Du S, et al. Blockade of Wnt/β-catenin pathway aggravated silica-induced lung inflammation through Tregs regulation on Th immune responses. Mediat Inflamm. 2016;2016:784. 10.1155/2016/6235614 PMC4812397 27069316

[36] Dong Y, Sun Q, Zhang X (2017). PD-1 and its ligands are important immune checkpoints in cancer. Oncotarget.

[37] Saleh R, Taha RZ, Sasidharan Nair V, Alajez NM, Elkord E (2019). PD-L1 blockade by atezolizumab downregulates signaling pathways associated with tumor growth, metastasis, and hypoxia in human triple negative breast cancer. Cancers.

[38] Castagnoli L, Cancila V, Cordoba-Romero SL, Faraci S, Talarico G, Belmonte B (2019). WNT signaling modulates PD-L1 expression in the stem cell compartment of triple-negative breast cancer. Oncogene.

[39] Castagnoli L, Tagliabue E, Pupa SM (2020). Inhibition of the Wnt signalling pathway: an avenue to control breast cancer aggressiveness. Int J Mol Sci.

[40] Uchino M, Kojima H, Wada K, Imada M, Onoda F, Satofuka H (2010). Nuclear β-catenin and CD44 upregulation characterize invasive cell populations in non-aggressive MCF-7 breast cancer cells. BMC Cancer.

[41] Cao T-T, Di Xiang B-LL, Huang T-X, Tan B-B, Zeng C-M, Wang Z-Y (2017). FZD7 is a novel prognostic marker and promotes tumor metastasis via WNT and EMT signaling pathways in esophageal squamous cell carcinoma. Oncotarget.

[42] Kirikoshi H, Sekihara H, Katoh M (2001). Up-regulation of Frizzled-7 (FZD7) in human gastric cancer. Int J Oncol.

[43] Shan S, Lv Q, Zhao Y, Liu C, Sun Y, Xi K (2015). Wnt/β-catenin pathway is required for epithelial to mesenchymal transition in CXCL12 over expressed breast cancer cells. Int J Clin Exp Pathol.

[44] Farahmand L, Merikhian P, Jalili N, Darvishi B, Majidzadeh-A K (2018). Significant role of MUC1 in development of resistance to currently existing anti-cancer therapeutic agents. Curr Cancer Drug Targets.

[45] Merikhian P, Ghadirian R, Farahmand L, Mansouri S, Majidzadeh-A K (2017). MUC1 induces tamoxifen resistance in estrogen receptor-positive breast cancer. Expert Rev Anticancer Ther.

[46] Li Y, Bharti A, Chen D, Gong J, Kufe D (1998). Interaction of glycogen synthase kinase 3β with the DF3/MUC1 carcinoma-associated antigen and β-catenin. Mol Cell Biol.

